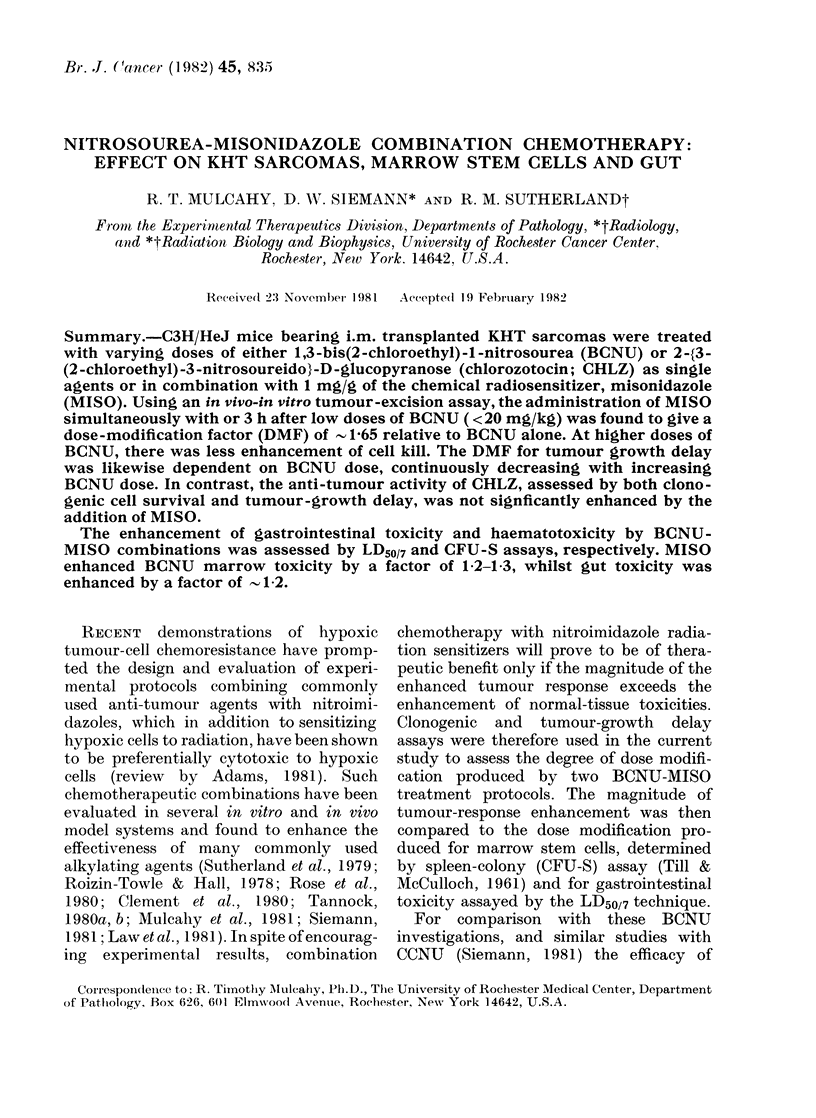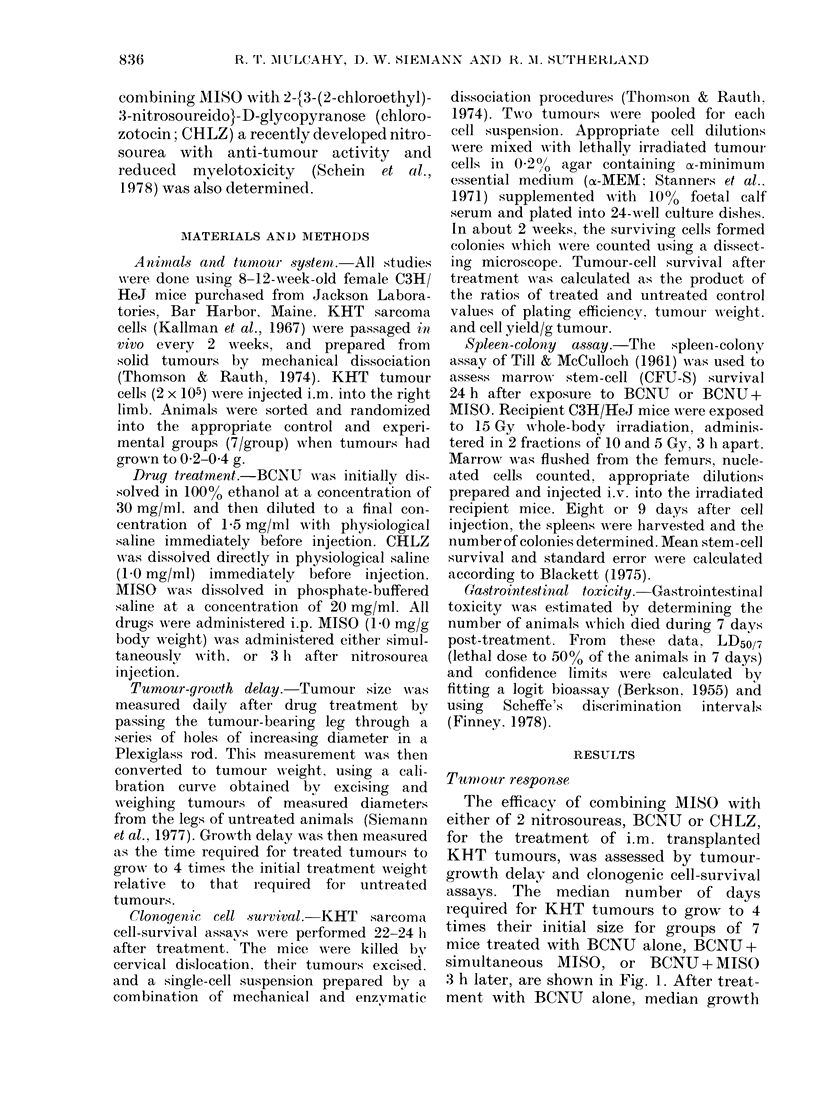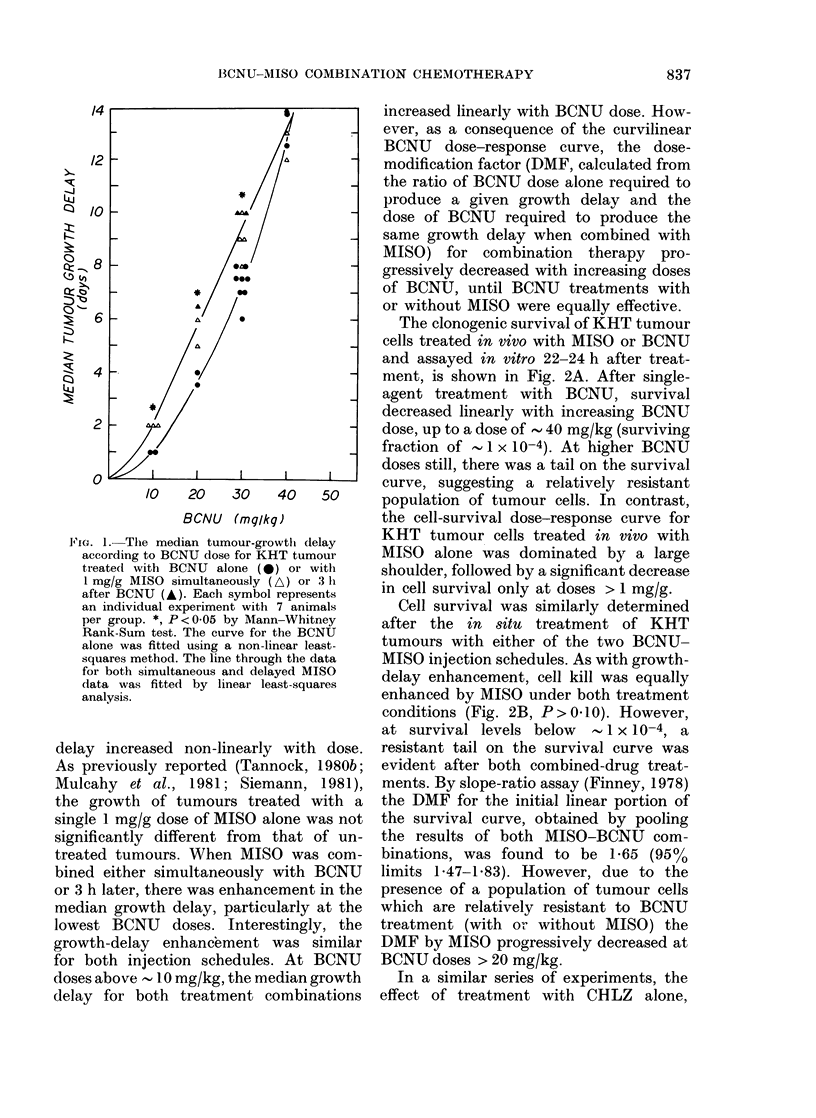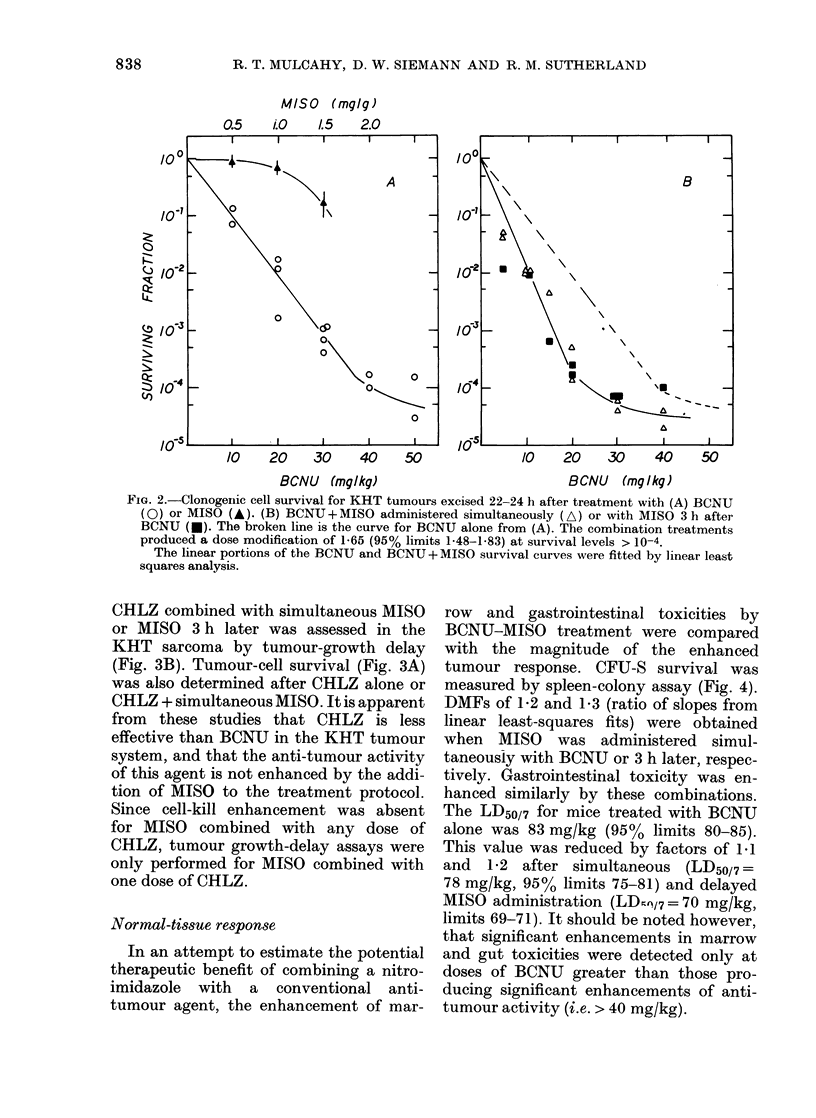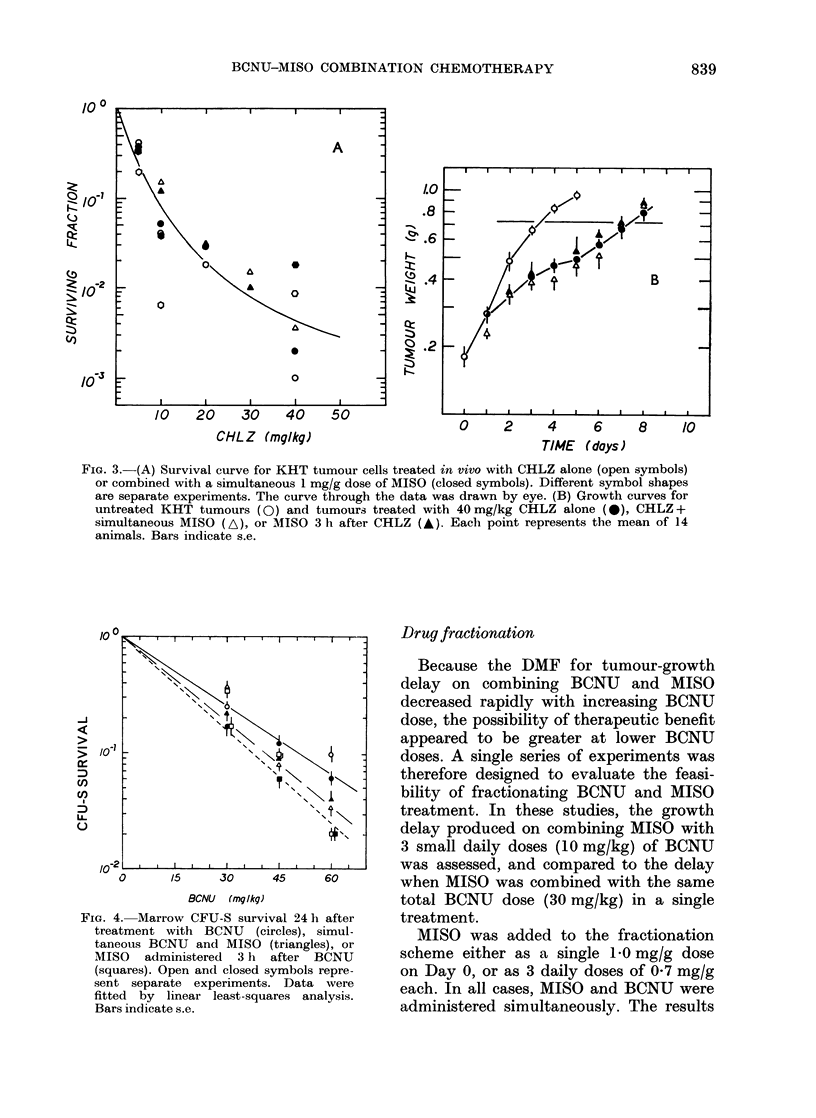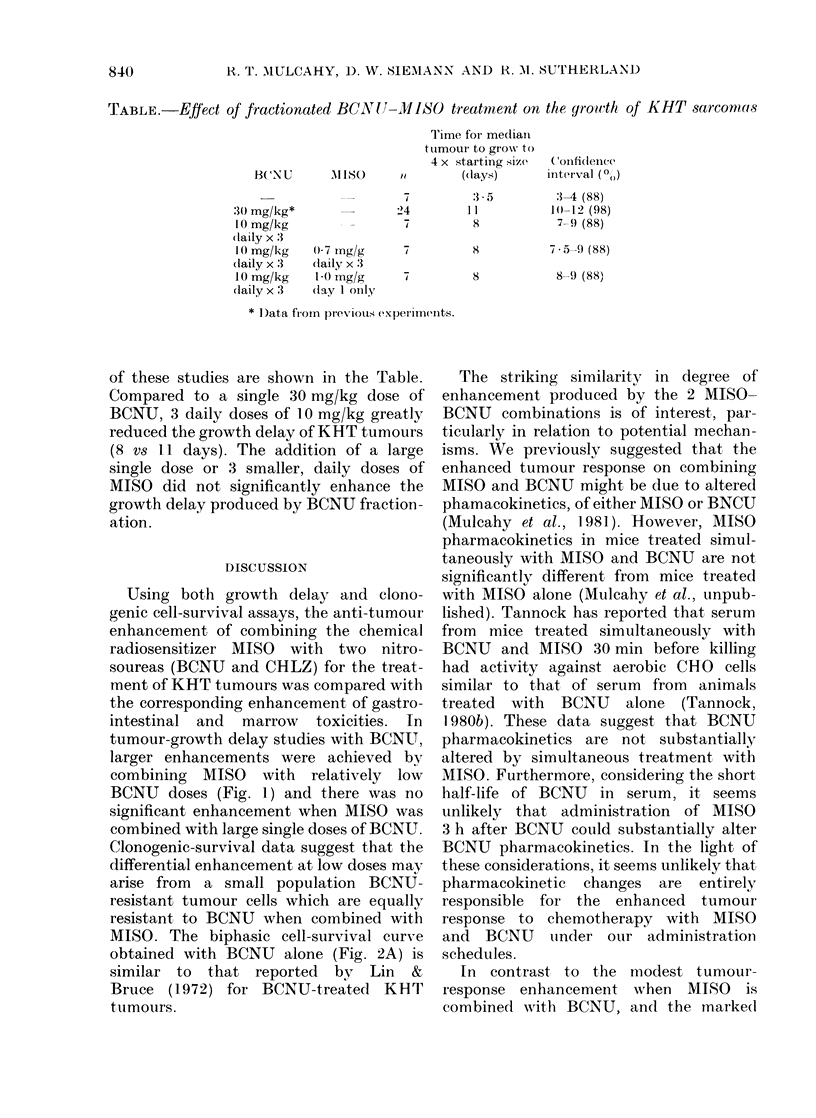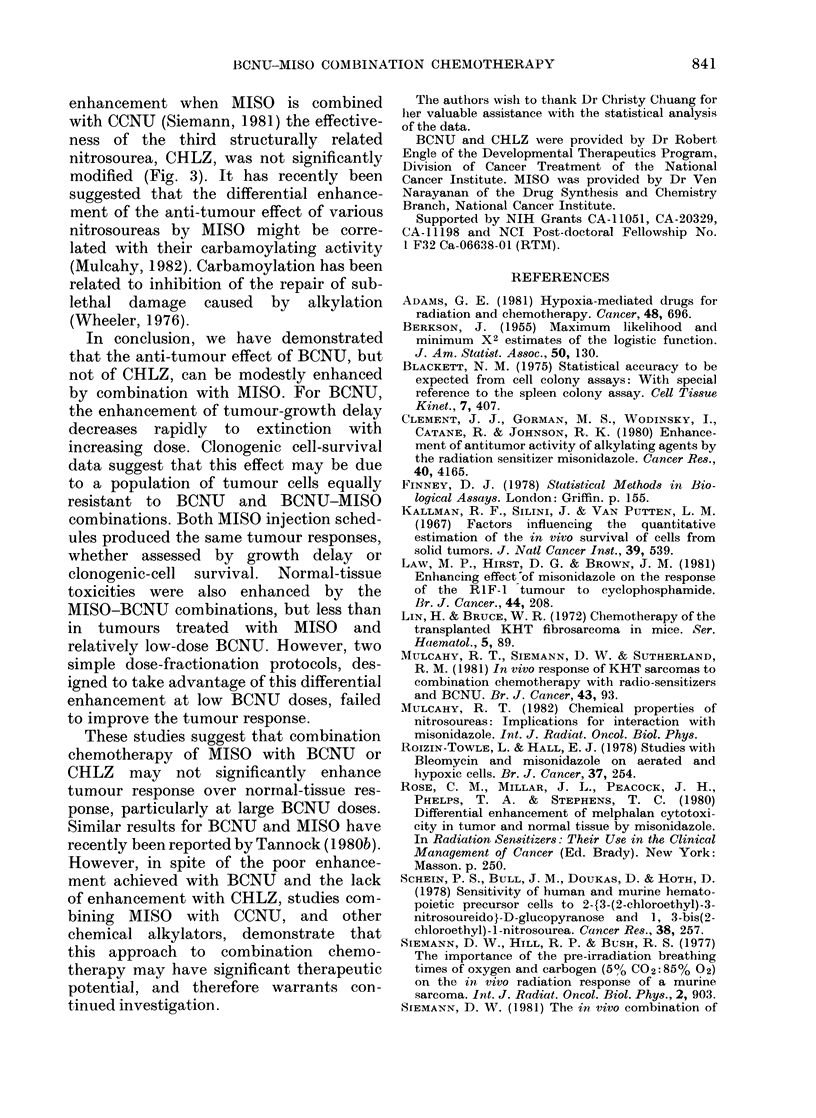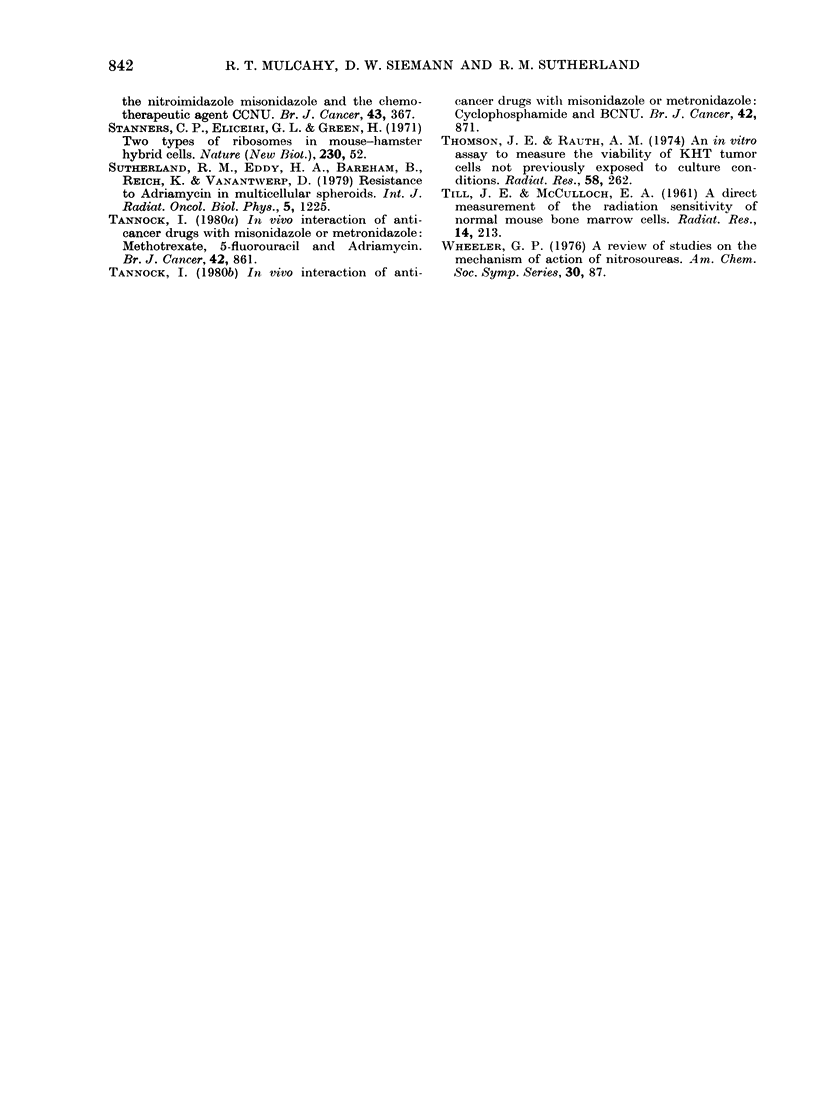# Nitrosourea-misonidazole combination chemotherapy: effect on KHT sarcomas, marrow stem cells and gut.

**DOI:** 10.1038/bjc.1982.135

**Published:** 1982-06

**Authors:** R. T. Mulcahy, D. W. Siemann, R. M. Sutherland

## Abstract

C3H/HeJ mice bearing i.m. transplanted KHT sarcomas were treated with varying doses of either 1,3-bis(2-chloroethyl)-1-nitrosourea (BCNU) or 2-[3-(2-chloroethyl)-3-nitrosoureido]-D-glucopyranose (chlorozotocin; CHLZ) as single agents or in combination with 1 mg/g of the chemical radiosensitizer, misonidazole (MISO). Using an in vivo-in vitro tumour-excision assay, the administration of MISO simultaneously with or 3 h after low doses of BCNU (less than 20 mg/kg) was found to give a dose-modification factor (DMF) of approximately 1.65 relative to BCNU alone. At higher doses of BCNU, there was less enhancement of cell kill. The DMF for tumour growth delay was likewise dependent on BCNU dose, continuously decreasing with increasing BCNU dose. In contrast, the anti-tumour activity of CHLZ, assessed by both clonogenic cell survival and tumour-growth delay, was not significantly enhanced by the addition of MISO. The enhancement of gastrointestinal toxicity and haematotoxicity by BCNU-MISO combinations was assessed by LD50/7 and CFU-S assays, respectively. MISO enhanced BCNU marrow toxicity by a factor of 1.2-1.3, whilst gut toxicity was enhanced by a factor of approximately 1.2.


Br. J. Cancer (1982) 45, 835

### NITROSOUREA-MISONIDAZOLE COMBINATION CHEMOTHERAPY: EFFECT ON KHT SARCOMAS, MARROW STEM CELLS AND GUT

R. T. MULCAHY, D. W. SIEMANN* AND R. M. SUTHERLAND†

*From the Experimental Therapeutics Division, Departments of Pathology, *†Radiology, and *†Radiation Biology and Biophysics, University of Rochester Cancer Center, Rochester, New York. 14642, U.S.A.*

Received 23 November 1981 Accepted 19 February 1982

**Summary.—C3H/HeJ mice bearing i.m. transplanted KHT sarcomas were treated with varying doses of either 1,3-bis(2-chloroethyl)-1-nitrosourea (BCNU) or 2-{3-(2-chloroethyl)-3-nitrosoureido}-D-glucopyranose (chlorozotocin; CHLZ) as single agents or in combination with 1 mg/g of the chemical radiosensitizer, misonidazole (MISO). Using an *in vivo-in vitro* tumour-excision assay, the administration of MISO simultaneously with or 3 h after low doses of BCNU (<20 mg/kg) was found to give a dose-modification factor (DMF) of ~1·65 relative to BCNU alone. At higher doses of BCNU, there was less enhancement of cell kill. The DMF for tumour growth delay was likewise dependent on BCNU dose, continuously decreasing with increasing BCNU dose. In contrast, the anti-tumour activity of CHLZ, assessed by both clonogenic cell survival and tumour-growth delay, was not signficantly enhanced by the addition of MISO.**

**The enhancement of gastrointestinal toxicity and haematotoxicity by BCNU-MISO combinations was assessed by $LD_{50/7}$ and CFU-S assays, respectively. MISO enhanced BCNU marrow toxicity by a factor of 1·2–1·3, whilst gut toxicity was enhanced by a factor of ~1·2.**

RECENT demonstrations of hypoxic tumour-cell chemoresistance have prompted the design and evaluation of experimental protocols combining commonly used anti-tumour agents with nitroimidazoles, which in addition to sensitizing hypoxic cells to radiation, have been shown to be preferentially cytotoxic to hypoxic cells (review by Adams, 1981). Such chemotherapeutic combinations have been evaluated in several *in vitro* and *in vivo* model systems and found to enhance the effectiveness of many commonly used alkylating agents (Sutherland *et al.*, 1979; Roizin-Towle & Hall, 1978; Rose *et al.*, 1980; Clement *et al.*, 1980; Tannock, 1980*a*, *b*; Mulcahy *et al.*, 1981; Siemann, 1981; Law *et al.*, 1981). In spite of encouraging experimental results, combination chemotherapy with nitroimidazole radiation sensitizers will prove to be of therapeutic benefit only if the magnitude of the enhanced tumour response exceeds the enhancement of normal-tissue toxicities. Clonogenic and tumour-growth delay assays were therefore used in the current study to assess the degree of dose modification produced by two BCNU-MISO treatment protocols. The magnitude of tumour-response enhancement was then compared to the dose modification produced for marrow stem cells, determined by spleen-colony (CFU-S) assay (Till & McCulloch, 1961) and for gastrointestinal toxicity assayed by the $LD_{50/7}$ technique.

For comparison with these BCNU investigations, and similar studies with CCNU (Siemann, 1981) the efficacy of

Correspondence to: R. Timothy Mulcahy, Ph.D., The University of Rochester Medical Center, Department of Pathology, Box 626, 601 Elmwood Avenue, Rochester, New York 14642, U.S.A.

combining MISO with 2-{3-(2-chloroethyl)-3-nitrosoureido}-D-glycopyranose (chlorozotocin; CHLZ) a recently developed nitrosourea with anti-tumour activity and reduced myelotoxicity (Schein *et al.*, 1978) was also determined.

### MATERIALS AND METHODS

*Animals and tumour system.*—All studies were done using 8–12-week-old female C3H/HeJ mice purchased from Jackson Laboratories, Bar Harbor, Maine. KHT sarcoma cells (Kallman *et al.*, 1967) were passaged *in vivo* every 2 weeks, and prepared from solid tumours by mechanical dissociation (Thomson & Rauth, 1974). KHT tumour cells ($2 \times 10^5$) were injected i.m. into the right limb. Animals were sorted and randomized into the appropriate control and experimental groups (7/group) when tumours had grown to 0·2–0·4 g.

*Drug treatment.*—BCNU was initially dissolved in 100% ethanol at a concentration of 30 mg/ml, and then diluted to a final concentration of 1·5 mg/ml with physiological saline immediately before injection. CHLZ was dissolved directly in physiological saline (1·0 mg/ml) immediately before injection. MISO was dissolved in phosphate-buffered saline at a concentration of 20 mg/ml. All drugs were administered i.p. MISO (1·0 mg/g body weight) was administered either simultaneously with, or 3 h after nitrosourea injection.

*Tumour-growth delay.*—Tumour size was measured daily after drug treatment by passing the tumour-bearing leg through a series of holes of increasing diameter in a Plexiglass rod. This measurement was then converted to tumour weight, using a calibration curve obtained by excising and weighing tumours of measured diameters from the legs of untreated animals (Siemann *et al.*, 1977). Growth delay was then measured as the time required for treated tumours to grow to 4 times the initial treatment weight relative to that required for untreated tumours.

*Clonogenic cell survival.*—KHT sarcoma cell-survival assays were performed 22–24 h after treatment. The mice were killed by cervical dislocation, their tumours excised, and a single-cell suspension prepared by a combination of mechanical and enzymatic dissociation procedures (Thomson & Rauth, 1974). Two tumours were pooled for each cell suspension. Appropriate cell dilutions were mixed with lethally irradiated tumour cells in 0·2% agar containing α-minimum essential medium (α-MEM; Stanners *et al.*, 1971) supplemented with 10% foetal calf serum and plated into 24-well culture dishes. In about 2 weeks, the surviving cells formed colonies which were counted using a dissecting microscope. Tumour-cell survival after treatment was calculated as the product of the ratios of treated and untreated control values of plating efficiency, tumour weight, and cell yield/g tumour.

*Spleen-colony assay.*—The spleen-colony assay of Till & McCulloch (1961) was used to assess marrow stem-cell (CFU-S) survival 24 h after exposure to BCNU or BCNU + MISO. Recipient C3H/HeJ mice were exposed to 15 Gy whole-body irradiation, administered in 2 fractions of 10 and 5 Gy, 3 h apart. Marrow was flushed from the femurs, nucleated cells counted, appropriate dilutions prepared and injected i.v. into the irradiated recipient mice. Eight or 9 days after cell injection, the spleens were harvested and the number of colonies determined. Mean stem-cell survival and standard error were calculated according to Blackett (1975).

*Gastrointestinal toxicity.*—Gastrointestinal toxicity was estimated by determining the number of animals which died during 7 days post-treatment. From these data, $LD_{50/7}$ (lethal dose to 50% of the animals in 7 days) and confidence limits were calculated by fitting a logit bioassay (Berkson, 1955) and using Scheffe's discrimination intervals (Finney, 1978).

### RESULTS

### *Tumour response*

The efficacy of combining MISO with either of 2 nitrosoureas, BCNU or CHLZ, for the treatment of i.m. transplanted KHT tumours, was assessed by tumour-growth delay and clonogenic cell-survival assays. The median number of days required for KHT tumours to grow to 4 times their initial size for groups of 7 mice treated with BCNU alone, BCNU + simultaneous MISO, or BCNU + MISO 3 h later, are shown in Fig. 1. After treatment with BCNU alone, median growth

FIG. 1.—The median tumour-growth delay according to BCNU dose for KHT tumour treated with BCNU alone (●) or with 1 mg/g MISO simultaneously (△) or 3 h after BCNU (▲). Each symbol represents an individual experiment with 7 animals per group. *, $P<0{\cdot}05$ by Mann–Whitney Rank-Sum test. The curve for the BCNU alone was fitted using a non-linear least-squares method. The line through the data for both simultaneous and delayed MISO data was fitted by linear least-squares analysis.

delay increased non-linearly with dose. As previously reported (Tannock, 1980*b*; Mulcahy *et al.*, 1981; Siemann, 1981), the growth of tumours treated with a single 1 mg/g dose of MISO alone was not significantly different from that of untreated tumours. When MISO was combined either simultaneously with BCNU or 3 h later, there was enhancement in the median growth delay, particularly at the lowest BCNU doses. Interestingly, the growth-delay enhancement was similar for both injection schedules. At BCNU doses above ~10 mg/kg, the median growth delay for both treatment combinations increased linearly with BCNU dose. However, as a consequence of the curvilinear BCNU dose–response curve, the dose-modification factor (DMF, calculated from the ratio of BCNU dose alone required to produce a given growth delay and the dose of BCNU required to produce the same growth delay when combined with MISO) for combination therapy progressively decreased with increasing doses of BCNU, until BCNU treatments with or without MISO were equally effective.

The clonogenic survival of KHT tumour cells treated *in vivo* with MISO or BCNU and assayed *in vitro* 22–24 h after treatment, is shown in Fig. 2A. After single-agent treatment with BCNU, survival decreased linearly with increasing BCNU dose, up to a dose of ~40 mg/kg (surviving fraction of $\sim 1\times10^{-4}$). At higher BCNU doses still, there was a tail on the survival curve, suggesting a relatively resistant population of tumour cells. In contrast, the cell-survival dose–response curve for KHT tumour cells treated *in vivo* with MISO alone was dominated by a large shoulder, followed by a significant decrease in cell survival only at doses $>1$ mg/g.

Cell survival was similarly determined after the *in situ* treatment of KHT tumours with either of the two BCNU–MISO injection schedules. As with growth-delay enhancement, cell kill was equally enhanced by MISO under both treatment conditions (Fig. 2B, $P>0{\cdot}10$). However, at survival levels below $\sim 1\times10^{-4}$, a resistant tail on the survival curve was evident after both combined-drug treatments. By slope-ratio assay (Finney, 1978) the DMF for the initial linear portion of the survival curve, obtained by pooling the results of both MISO–BCNU combinations, was found to be 1·65 (95% limits 1·47–1·83). However, due to the presence of a population of tumour cells which are relatively resistant to BCNU treatment (with or without MISO) the DMF by MISO progressively decreased at BCNU doses $>20$ mg/kg.

In a similar series of experiments, the effect of treatment with CHLZ alone,

FIG. 2.—Clonogenic cell survival for KHT tumours excised 22–24 h after treatment with (A) BCNU (○) or MISO (▲). (B) BCNU+MISO administered simultaneously (△) or with MISO 3 h after BCNU (■). The broken line is the curve for BCNU alone from (A). The combination treatments produced a dose modification of 1·65 (95% limits 1·48–1·83) at survival levels $> 10^{-4}$.

The linear portions of the BCNU and BCNU+MISO survival curves were fitted by linear least squares analysis.

CHLZ combined with simultaneous MISO or MISO 3 h later was assessed in the KHT sarcoma by tumour-growth delay (Fig. 3B). Tumour-cell survival (Fig. 3A) was also determined after CHLZ alone or CHLZ + simultaneous MISO. It is apparent from these studies that CHLZ is less effective than BCNU in the KHT tumour system, and that the anti-tumour activity of this agent is not enhanced by the addition of MISO to the treatment protocol. Since cell-kill enhancement was absent for MISO combined with any dose of CHLZ, tumour growth-delay assays were only performed for MISO combined with one dose of CHLZ.

*Normal-tissue response*

In an attempt to estimate the potential therapeutic benefit of combining a nitroimidazole with a conventional antitumour agent, the enhancement of marrow and gastrointestinal toxicities by BCNU–MISO treatment were compared with the magnitude of the enhanced tumour response. CFU-S survival was measured by spleen-colony assay (Fig. 4). DMFs of 1·2 and 1·3 (ratio of slopes from linear least-squares fits) were obtained when MISO was administered simultaneously with BCNU or 3 h later, respectively. Gastrointestinal toxicity was enhanced similarly by these combinations. The $LD_{50/7}$ for mice treated with BCNU alone was 83 mg/kg (95% limits 80–85). This value was reduced by factors of 1·1 and 1·2 after simultaneous ($LD_{50/7}$ = 78 mg/kg, 95% limits 75–81) and delayed MISO administration ($LD_{50/7}$ = 70 mg/kg, limits 69–71). It should be noted however, that significant enhancements in marrow and gut toxicities were detected only at doses of BCNU greater than those producing significant enhancements of antitumour activity (*i.e.* > 40 mg/kg).

FIG. 3.—(A) Survival curve for KHT tumour cells treated *in vivo* with CHLZ alone (open symbols) or combined with a simultaneous 1 mg/g dose of MISO (closed symbols). Different symbol shapes are separate experiments. The curve through the data was drawn by eye. (B) Growth curves for untreated KHT tumours (○) and tumours treated with 40 mg/kg CHLZ alone (●), CHLZ + simultaneous MISO (△), or MISO 3 h after CHLZ (▲). Each point represents the mean of 14 animals. Bars indicate s.e.

FIG. 4.—Marrow CFU-S survival 24 h after treatment with BCNU (circles), simultaneous BCNU and MISO (triangles), or MISO administered 3 h after BCNU (squares). Open and closed symbols represent separate experiments. Data were fitted by linear least-squares analysis. Bars indicate s.e.

*Drug fractionation*

Because the DMF for tumour-growth delay on combining BCNU and MISO decreased rapidly with increasing BCNU dose, the possibility of therapeutic benefit appeared to be greater at lower BCNU doses. A single series of experiments was therefore designed to evaluate the feasibility of fractionating BCNU and MISO treatment. In these studies, the growth delay produced on combining MISO with 3 small daily doses (10 mg/kg) of BCNU was assessed, and compared to the delay when MISO was combined with the same total BCNU dose (30 mg/kg) in a single treatment.

MISO was added to the fractionation scheme either as a single 1·0 mg/g dose on Day 0, or as 3 daily doses of 0·7 mg/g each. In all cases, MISO and BCNU were administered simultaneously. The results

TABLE.—*Effect of fractionated BCNU–MISO treatment on the growth of KHT sarcomas*

| BCNU | MISO | n | Time for median tumour to grow to 4 × starting size (days) | Confidence interval (%) |
|---|---|---|---|---|
| — | — | 7 | 3·5 | 3–4 (88) |
| 30 mg/kg* | — | 24 | 11 | 10–12 (98) |
| 10 mg/kg daily × 3 | — | 7 | 8 | 7–9 (88) |
| 10 mg/kg daily × 3 | 0·7 mg/g daily × 3 | 7 | 8 | 7·5–9 (88) |
| 10 mg/kg daily × 3 | 1·0 mg/g day 1 only | 7 | 8 | 8–9 (88) |

* Data from previous experiments.

of these studies are shown in the Table. Compared to a single 30 mg/kg dose of BCNU, 3 daily doses of 10 mg/kg greatly reduced the growth delay of KHT tumours (8 *vs* 11 days). The addition of a large single dose or 3 smaller, daily doses of MISO did not significantly enhance the growth delay produced by BCNU fractionation.

### DISCUSSION

Using both growth delay and clonogenic cell-survival assays, the anti-tumour enhancement of combining the chemical radiosensitizer MISO with two nitrosoureas (BCNU and CHLZ) for the treatment of KHT tumours was compared with the corresponding enhancement of gastrointestinal and marrow toxicities. In tumour-growth delay studies with BCNU, larger enhancements were achieved by combining MISO with relatively low BCNU doses (Fig. 1) and there was no significant enhancement when MISO was combined with large single doses of BCNU. Clonogenic-survival data suggest that the differential enhancement at low doses may arise from a small population BCNU-resistant tumour cells which are equally resistant to BCNU when combined with MISO. The biphasic cell-survival curve obtained with BCNU alone (Fig. 2A) is similar to that reported by Lin & Bruce (1972) for BCNU-treated KHT tumours.

The striking similarity in degree of enhancement produced by the 2 MISO–BCNU combinations is of interest, particularly in relation to potential mechanisms. We previously suggested that the enhanced tumour response on combining MISO and BCNU might be due to altered phamacokinetics, of either MISO or BNCU (Mulcahy *et al.*, 1981). However, MISO pharmacokinetics in mice treated simultaneously with MISO and BCNU are not significantly different from mice treated with MISO alone (Mulcahy *et al.*, unpublished). Tannock has reported that serum from mice treated simultaneously with BCNU and MISO 30 min before killing had activity against aerobic CHO cells similar to that of serum from animals treated with BCNU alone (Tannock, 1980*b*). These data suggest that BCNU pharmacokinetics are not substantially altered by simultaneous treatment with MISO. Furthermore, considering the short half-life of BCNU in serum, it seems unlikely that administration of MISO 3 h after BCNU could substantially alter BCNU pharmacokinetics. In the light of these considerations, it seems unlikely that pharmacokinetic changes are entirely responsible for the enhanced tumour response to chemotherapy with MISO and BCNU under our administration schedules.

In contrast to the modest tumour-response enhancement when MISO is combined with BCNU, and the marked enhancement when MISO is combined with CCNU (Siemann, 1981) the effectiveness of the third structurally related nitrosourea, CHLZ, was not significantly modified (Fig. 3). It has recently been suggested that the differential enhancement of the anti-tumour effect of various nitrosoureas by MISO might be correlated with their carbamoylating activity (Mulcahy, 1982). Carbamoylation has been related to inhibition of the repair of sublethal damage caused by alkylation (Wheeler, 1976).

In conclusion, we have demonstrated that the anti-tumour effect of BCNU, but not of CHLZ, can be modestly enhanced by combination with MISO. For BCNU, the enhancement of tumour-growth delay decreases rapidly to extinction with increasing dose. Clonogenic cell-survival data suggest that this effect may be due to a population of tumour cells equally resistant to BCNU and BCNU–MISO combinations. Both MISO injection schedules produced the same tumour responses, whether assessed by growth delay or clonogenic-cell survival. Normal-tissue toxicities were also enhanced by the MISO–BCNU combinations, but less than in tumours treated with MISO and relatively low-dose BCNU. However, two simple dose-fractionation protocols, designed to take advantage of this differential enhancement at low BCNU doses, failed to improve the tumour response.

These studies suggest that combination chemotherapy of MISO with BCNU or CHLZ may not significantly enhance tumour response over normal-tissue response, particularly at large BCNU doses. Similar results for BCNU and MISO have recently been reported by Tannock (1980*b*). However, in spite of the poor enhancement achieved with BCNU and the lack of enhancement with CHLZ, studies combining MISO with CCNU, and other chemical alkylators, demonstrate that this approach to combination chemotherapy may have significant therapeutic potential, and therefore warrants continued investigation.

The authors wish to thank Dr Christy Chuang for her valuable assistance with the statistical analysis of the data.

BCNU and CHLZ were provided by Dr Robert Engle of the Developmental Therapeutics Program, Division of Cancer Treatment of the National Cancer Institute. MISO was provided by Dr Ven Narayanan of the Drug Synthesis and Chemistry Branch, National Cancer Institute.

Supported by NIH Grants CA-11051, CA-20329, CA-11198 and NCI Post-doctoral Fellowship No. 1 F32 Ca-06638-01 (RTM).

### REFERENCES

Adams, G. E. (1981) Hypoxia-mediated drugs for radiation and chemotherapy. *Cancer*, **48**, 696.

Berkson, J. (1955) Maximum likelihood and minimum $X^2$ estimates of the logistic function. *J. Am. Statist. Assoc.*, **50**, 130.

Blackett, N. M. (1975) Statistical accuracy to be expected from cell colony assays: With special reference to the spleen colony assay. *Cell Tissue Kinet.*, **7**, 407.

Clement, J. J., Gorman, M. S., Wodinsky, I., Catane, R. & Johnson, R. K. (1980) Enhancement of antitumor activity of alkylating agents by the radiation sensitizer misonidazole. *Cancer Res.*, **40**, 4165.

Finney, D. J. (1978) *Statistical Methods in Biological Assays*. London: Griffin. p. 155.

Kallman, R. F., Silini, J. & Van Putten, L. M. (1967) Factors influencing the quantitative estimation of the *in vivo* survival of cells from solid tumors. *J. Natl Cancer Inst.*, **39**, 539.

Law, M. P., Hirst, D. G. & Brown, J. M. (1981) Enhancing effect of misonidazole on the response of the RIF-1 tumour to cyclophosphamide. *Br. J. Cancer.*, **44**, 208.

Lin, H. & Bruce, W. R. (1972) Chemotherapy of the transplanted KHT fibrosarcoma in mice. *Ser. Haematol.*, **5**, 89.

Mulcahy, R. T., Siemann, D. W. & Sutherland, R. M. (1981) *In vivo* response of KHT sarcomas to combination chemotherapy with radio-sensitizers and BCNU. *Br. J. Cancer*, **43**, 93.

Mulcahy, R. T. (1982) Chemical properties of nitrosoureas: Implications for interaction with misonidazole. *Int. J. Radiat. Oncol. Biol. Phys.*

Roizin-Towle, L. & Hall, E. J. (1978) Studies with Bleomycin and misonidazole on aerated and hypoxic cells. *Br. J. Cancer*, **37**, 254.

Rose, C. M., Millar, J. L., Peacock, J. H., Phelps, T. A. & Stephens, T. C. (1980) Differential enhancement of melphalan cytotoxicity in tumor and normal tissue by misonidazole. In *Radiation Sensitizers: Their Use in the Clinical Management of Cancer* (Ed. Brady). New York: Masson. p. 250.

Schein, P. S., Bull, J. M., Doukas, D. & Hoth, D. (1978) Sensitivity of human and murine hematopoietic precursor cells to 2-{3-(2-chloroethyl)-3-nitrosoureido}-D-glucopyranose and 1, 3-bis(2-chloroethyl)-1-nitrosourea. *Cancer Res.*, **38**, 257.

Siemann, D. W., Hill, R. P. & Bush, R. S. (1977) The importance of the pre-irradiation breathing times of oxygen and carbogen (5% $CO_2$:85% $O_2$) on the *in vivo* radiation response of a murine sarcoma. *Int. J. Radiat. Oncol. Biol. Phys.*, **2**, 903.

Siemann, D. W. (1981) The *in vivo* combination of the nitroimidazole misonidazole and the chemotherapeutic agent CCNU. *Br. J. Cancer*, **43**, 367.

STANNERS, C. P., ELICEIRI, G. L. & GREEN, H. (1971) Two types of ribosomes in mouse–hamster hybrid cells. *Nature* (*New Biol.*), **230**, 52.

SUTHERLAND, R. M., EDDY, H. A., BAREHAM, B., REICH, K. & VANANTWERP, D. (1979) Resistance to Adriamycin in multicellular spheroids. *Int. J. Radiat. Oncol. Biol. Phys.*, **5**, 1225.

TANNOCK, I. (1980*a*) *In vivo* interaction of anticancer drugs with misonidazole or metronidazole: Methotrexate, 5-fluorouracil and Adriamycin. *Br. J. Cancer*, **42**, 861.

TANNOCK, I. (1980*b*) *In vivo* interaction of anticancer drugs with misonidazole or metronidazole: Cyclophosphamide and BCNU. *Br. J. Cancer*, **42**, 871.

THOMSON, J. E. & RAUTH, A. M. (1974) An *in vitro* assay to measure the viability of KHT tumor cells not previously exposed to culture conditions. *Radiat. Res.*, **58**, 262.

TILL, J. E. & MCCULLOCH, E. A. (1961) A direct measurement of the radiation sensitivity of normal mouse bone marrow cells. *Radiat. Res.*, **14**, 213.

WHEELER, G. P. (1976) A review of studies on the mechanism of action of nitrosoureas. *Am. Chem. Soc. Symp. Series*, **30**, 87.